# Current Trends in Bioaugmentation Tools for Bioremediation: A Critical Review of Advances and Knowledge Gaps

**DOI:** 10.3390/microorganisms11030710

**Published:** 2023-03-09

**Authors:** Olga Muter

**Affiliations:** Faculty of Biology, University of Latvia, LV-1004 Riga, Latvia; olga.mutere@lu.lv

**Keywords:** bioaugmentation, contamination, microbial succession

## Abstract

Bioaugmentation is widely used in soil bioremediation, wastewater treatment, and air biofiltration. The addition of microbial biomass to contaminated areas can considerably improve their biodegradation performance. Nevertheless, analyses of large data sets on the topic available in literature do not provide a comprehensive view of the mechanisms responsible for inoculum-assisted stimulation. On the one hand, there is no universal mechanism of bioaugmentation for a broad spectrum of environmental conditions, contaminants, and technology operation concepts. On the other hand, further analyses of bioaugmentation outcomes under laboratory conditions and in the field will strengthen the theoretical basis for a better prediction of bioremediation processes under certain conditions. This review focuses on the following aspects: (i) choosing the source of microorganisms and the isolation procedure; (ii) preparation of the inoculum, e.g., cultivation of single strains or consortia, adaptation; (iii) application of immobilised cells; (iv) application schemes for soil, water bodies, bioreactors, and hydroponics; and (v) microbial succession and biodiversity. Reviews of recent scientific papers dating mostly from 2022–2023, as well as our own long-term studies, are provided here.

## 1. Introduction

Bioremediation encompasses a broad range of environmental biotechnologies, which require multidisciplinary approaches through implementation of innovative tools to the natural biological processes occurring in soil, water, and air. The addition of microbial biomass (bacteria, fungi, and their secreted enzymes) to contaminated areas, i.e., the process of bioaugmentation, can be adapted to the green environment and can notably improve an area’s pollutant removal efficiency (RE), as well as reduce their removal time and costs [1]. However, bioaugmentation under controlled conditions in the field remains challenging, due to the biodiversity of a whole system, competition between microbial agents and indigenous microorganisms, substrate competition, climatic conditions, remediation cycles, and other factors. To select an optimal bioaugmentation strategy, further studies on the interactions of different functional bacteria related to their resistance to multiple stress factors, enzyme activity and system robustness are needed [2]. Rigorous research and critical analyses of available databases, as well as the incorporation of genetic engineering, nanotechnology, and systems biology can bring bioremediation to a more advanced level [3].

Bioaugmentation is a site-specific approach. Thus, recent research publications and reviews on bioaugmentation have focused on the following aspects: thermophilic reductive dechlorination [4,5]; the psychrophilic treatment of groundwaters [6]; microbially induced calcium carbonate precipitation techniques to mitigate the wind-induced erosion of calcareous desert sand [7]; hydrocarbon biodegradation in freshwater sediments from historically contaminated lakes [8]; the bacterial remediation of pesticide-polluted soils [3], emerging trends in the remediation of organic contaminated soils as a whole [9]; mechanisms of microbial activity in heavy metal removal [10]; comparisons of autochthonous and allochthonous bioaugmentation [11]; the stimulation of plant growth in bioaugmented hydroponic systems [12]; and the bioaugmentation of wastewaters (WWs) with yeast in the presence of antimicrobials [13], among others.

The aim of this review was to gather different aspects related to bioaugmentation approaches. Bioaugmentation has received increasing interest from the scientific community over the last five years. Indeed, a total of 160 articles and 12 reviews on this topic were published in 2017, while in 2022 these numbers had increased to 1447 and 148, respectively (database SCOPUS, keyword “bioaugmentation”). In this respect, to avoid possible repetitions, this review focused mostly on the scientific articles and review papers published in 2022–2023, as well as the authors’ own research results.

## 2. Sources of Microorganisms for Bioaugmentation

There are abundant data available on the variations and successions of microbial communities upon bioaugmentation-assisted remediation where the introduced microbial “agent” disappears during the remediation process. However, positive effects on biodegradation dynamics have been detected.

Our recent studies have demonstrated a dominant abundance of the Proteobacteria and Firmicutes phyla in various biodegradation processes assisted by autochthonous and allochthonous bioaugmentation.

In a study of the bioremediation of hydrocarbon-contaminated lake sediments, a 32-day batch incubation in (i) biostimulated (N), (ii) biostimulated and bioaugmented (NB), and (iii) unamended (K) sets resulted in an increasing abundance of Proteobacteria from 48.8% in the raw sediments to 59.3%, 58.0%, and 61.7%, respectively, mainly due to an increase in Betaproteobacteria. The relative abundance of the *Pseudomonas* genus (Gammaproteobacteria) was the highest in the (N) set at 35.8%, compared to 17.5% and 21.0% for the (K) and (NB) sets, respectively. The genera that dominated in the inoculum (allochthonous) were identified as Citrobacter (29.5%), Klebsiella (24.5%), Pseudomonas (15.5%), and Aeromonas (11.3%). The concentration of hydrocarbons in the lake sediments during the 32-day incubation decreased on average from 465 mg/kg to 165 mg/kg and 117.5 mg/kg in the (N) and (NB) sets, respectively [8].

Another study focused on the effect of two allochthonous bacterial consortia on the growth of *Mentha aquatica* in a hydroponic system. After the 47-day hydroponic greenhouse experiment, the structure of the bacterial communities attached to expanded clay pellets was represented mostly by Proteobacteria at the phylum level (80–90%). Bioaugmentation provided significant (*p* < 0.05) stimulation for the growth of *M. aquatica*, although the metagenome analysis of the rhizosphere did not reveal any abundance of bacterial strains, which were introduced into the hydroponic media at the beginning of the experiment [12].

The bioaugmentation of municipal sewage sludge/straw substrate with an autochthonous bacterial consortium resulted in a marked shift in the microbial community structure. Particularly, the raw sewage sludge contained Firmicutes/Proteobacteria/Actinobacteria at levels of 4.4%/60.2%/26.5%, respectively, while the inoculum for bioaugmentation, which was prepared using a selective broth, contained these phyla in proportions of 92.13%/1.7%/5.1%, respectively. After 16 days of batch incubation, the proportions varied as follows: (32.7–53.8%)/(30.6–54.3%)/(5.3–13.7%). All treated samples were characterised by an increased abundance of Firmicutes. Yet, an increased abundance of ungrouped reads of *Pseudomonas putida* was detected in all bioaugmented sets; however, it was not detected in the inoculum. Compared with non-bioaugmented sets, the combination of a wheat straw amendment to a sewage sludge with bioaugmentation showed the highest and most stable microbial respiration intensity, the lowest ammonia emissions, and the highest stimulation effect on cress seedling growth [14]. More recent studies have also shown a positive effect of bioaugmentation on manure composting, which influenced the bacterial response, matter transformation, and metal immobilisation [15].

The effect of the bioaugmentation of activated sludge with viable brewing spent yeast biomass on microbial community structure was studied in the presence of benzalkonium chloride. The added yeast biomass remained viable during 10-day treatment and reduced an inhibitory effect of BAC on *Bacilli* in activated sludge. Yet, the bioaugmentation stimulated bacterial growth and microbial respiration. At the phylum level, two dominant taxa, i.e., Firmicutes and Proteobacteria, were found in the activated sludge, with their abundance in the control (non-incubated) and all incubated samples ranging between 27–35% and 22–36%, respectively [13].

Recently, Ref. [16] reported that Proteobacteria, Firmicutes, Bacteroidetes, Actinobacteria, and Acidobacteria were the dominant phyla during the biodegradation of crude oil. Some key enzymes related to the biodegradation of petroleum products have been detected in *Bacillus megaterium* (alkane hydroxylase, catechol 1,2-dioxygenase, protocatechol 3,4-dioxygenase), *Bacillus pumilus* (esterases and lipase), *Pseudomonas aeruginosa* (catechol 1,2-dioxygenase, protocatechol 3,4-dioxygenase), and *Stenotrophomonas maltophilia* (catechol 2,3-dioxygenase) [17]. The use of manganese-oxidising *Pseudomonas* sp. QJX-1 with humic acids as the sole carbon source has been proposed for the removal of pharmaceuticals (caffeine) from drinking water via sand filtration [18]. Furthermore, *Rhodococcus* spp. are known to play an important role in the biodegradation of organic contaminants, as well as in the recovery of the nitrification performance in the presence of antibacterial agents in activated sludge and other processes [19,20,21]. The catabolic activity of rhodococci involves catabolizing short- and long-chain alkanes, as well as aromatic (halogenated and nitro-substituted), heterocyclic, and polycyclic aromatic compounds. The high adaptability of rhodococci with respect to substrates has previously been reviewed by [22], with an emphasis on hyperrecombination evolutionary strategies. Linear plasmids in the large *Rhodococcus* genomes store multiple copies of many biodegradative genes [22]. Bacteria of the genera *Pseudomonas*, *Bacillus*, and *Rhodococcus* can be found in a broad range of ecosystems exhibiting extraordinary activities in the breakdown of natural pollutants and xenobiotics and taking part in microbial consortia and/or endophytic cooperation.

Although the overall microbial community structure in organics-polluted sites commonly depends on the geographic location [23], some bacterial genera are often predominant.

On the one hand, *Bacillus* spp., *Pseudomonas* spp., and *Rhodococcus* spp. appear to be dominant because of microbial succession upon biodegradation. On the other hand, researchers frequently use these bacteria as an inoculum for bioaugmentation [1,24].

Searching for “*Pseudomonas* bioaugmentation”, “*Bacillus* bioaugmentation”, and “*Rhodococcus* bioaugmentation” in the SCOPUS database for studies published in 2022 revealed 52, 37, and 23 sources, respectively. Some recent studies are summarised in Table 1.

## 3. Obtaining Microorganism Degraders: Sources and Methods

Functional consortia of microorganisms with a high degradation activity can be isolated from contaminated sites, agricultural waste, activated sludge, and other sources which are characterised by a relatively high microbial biodiversity. Specific conditions chosen for the isolation and further cultivation of these microorganisms include a selective pressure, which allows the formation of a distinct subpopulation of bacteria. Alternatively, intact nutrient- and microorganism-rich substrates such as compost and activated sludge can be applied to bioaugmentation (without isolation procedures). Furthermore, genetically modified organisms can also be used. Some of following bioaugmentation sources were reported in 2022:

-Vermicompost allowed the enrichment of resilient and degrading microorganisms which could be extracted through an aerated aqueous extraction process. The authors suggested a simple and fast isolation of microbial consortia by aerated aqueous extraction [42]. The application of vermicompost on the moderately weathered soil polluted with heavy linear alkanes resulted in five-fold and two-fold increases in the amounts of available phosphorus (P) and exchangeable potassium (K), respectively. Hydrocarbon degradation was increased by up to 34.4% relative to the control [43].

-Activated sludge. The effect of the introduction of exogenous activated sludge on the activity and composition of the microbial consortium carrying out the nitritation–anammox process in a sequencing batch bioreactor was investigated by [44]. The addition of exogenous activated sludge after the stable nitrogen removal mode was reached (day 53) increased the efficiency of nitrogen removal by 21–35%, and this difference was maintained until the end of the experiment (90 days) [44].

-Plant growth-promoting rhizobacteria (PGPR) as a bioaugmentation tool for treating soils contaminated by hydrocarbons [45], polyesters [46], and heavy metals [47]. PGPR produce multiple types of biosurfactants and diverse oxygenases in variable bacterial species, e.g., *Pseudomonas*, *Acinetobacter*, *Mycobacterium*, *Haemophilus*, *Rhodococcus*, *Paenibacillus*, and *Ralstonia* [45,48]. A recent study of the effect of the rhizobacteria *Bacillus subtilis* on cadmium bioavailability and distribution in soil planted with ryegrass (*Lolium multiflorum* L.) demonstrated a reduction in Cd bioavailability by 39.1%, followed by alterations in the microbial community structure, e.g., enrichment of Proteobacteria [47]. The effect of rice assisted with a PGPR consortium (three isolates of *Bacillus* sp., *Agrobacterium* sp.) on the remediation of a multi-compound (i.e., di (2-ethylhexyl) phthalate, Cd, and Zn)-contaminated site was recently studied by [46]. The treatment resulted in the removal of di (2-ethylhexyl) phthalate, Cd, and Zn by 86,1%, 76.0%, and 92.2%, respectively, within 30 days [46].

-Genetic bioaugmentation. Genetically modified organisms have also been shown to improve stability and resistance to environmental stressors, and their prolonged viability results in greater effectiveness [49,50,51]. Bioaugmentation with *Pseudomonas putida* KT2440, harbouring the transferrable triclocarban-catabolic plasmid pDCA-1-*gfp-tccA2*, rapidly converted 50 μM triclocarban in WW into 3,4-dichloroaniline and 4-chloroaniline, which were further mineralised more easily [52]. In genetic bioaugmentation for pollutant removal, a donor bacterium harbouring a catabolic plasmid will transfer the plasmid to a recipient cell (transconjugant) and both the donor and transconjugant can express degradation genes for the removal of the contaminant. Varner et al. [53] explored the effect of the ecological growth strategies of plasmid donors and recipients on the conjugation and naphthalene degradation of two PAH-degrading plasmids, pNL1 and NAH7 [53]. Bokade et al. [3] reviewed the suggested mobile genetic elements mediating the horizontal transfer of pesticide degradation genes, i.e., plasmids, transposons, genomic islands, transcription sequences, and integron gene cassettes.

Contaminated sites can act as a repository of highly adapted diverse populations which must be harnessed to score different degraders. Bokade et al. [3] reviewed different enrichment approaches employed in the isolation of microorganisms from diverse environments, i.e., magnetic separation, differential centrifugation, micromanipulation, dilution to extinction, concentration to extinction, toxicity to extinction, heat pretreatment, and dilution-to-stimulation/extinction. Enrichment techniques offer an effective strategy for selectively isolating the microorganisms of interest. In this technique, specific environmental conditions are simulated to increase the abundance of organisms to a detectable level. Usually, this method involves the use of specific growth media and conditions that favour the growth of a specific microorganism over others. Several techniques including micromanipulation, magnetic separation, differential centrifugation, dilution-to-extinction, concentration-to-extinction, and toxicity-to-extinction have been applied for the enrichment and isolation of specific degrader microorganisms, depending on the intended purpose [3].

Functional consortia can be developed by collecting different isolates with certain target properties. More specifically, [54] developed the following microbial consortium for composting: protein-degrading bacteria (*Brevibacillus brevis*), starch-degrading bacteria (*Acinetobacter johnonii*), ammonia-oxidising bacteria (*Ureibacillus terrenus*), oil degraders (*Aneurinibacillus thermoaerophilus*, *Bacillus hisahii*, *Candida tropicalis*), and mixed lignocellulose-degrading microorganisms [54]. In a study using an aquaculture WW treatment, an inoculum consisting of algae and bacteria was tested. This consortium provided the most compact biofloc structure (0.59 g/L), high settleability (71.91%), and a large particle diameter (4.25 mm) [30]. The mixed salt-tolerant bacteria system composed of ammonia-, nitrite-, and nitrate–nitrogen-utilising bacteria was artificially constructed for the salt-tolerant aerobic granular sludge. The flocculent consortium was aggregated by *Aspergillus tubingensis* mycelium pellet regions [2].

The effects of bioaugmentation by single cultures and consortia on the biodegradation of highly chlorinated compounds, i.e., decachlorobiphenyl (PCB-209), were compared by [29]. The application of a consortium resulted in the highest PCB-209 removal potential [29]. Another study showed that consortia and individual strains had similar crude oil-degrading capacities [11]. Moreover, [37] reported that the oil biodegradation efficiency of single strains and consortia (*Actinotalea ferrariae*, *Arthrobacter ginsengisoli*, *Dietzia cinnamea*, *Dietzia papillomatosis*, and *Pseudomonas songnenensis*) was similar, and that the REs of bioaugmented and non-bioaugmented sets in an oil-saturated desert soil after a 6-month experiment were also similar, exhibiting similar predominant bacterial species, e.g., *Ar. ginsengisoli* [37].

Regarding synergistic, syntrophic, antagonistic, neutral, or other types of microbial interrelations, Foster and Bell suggested that adaptation to other microbial species typically results in competitive rather than cooperative phenotypes. The positive effects in one direction, where one species gains at the expense of another, i.e., through predator–prey-like interactions, were not excluded [55].

## 4. Preparation of the Inoculum

The adaptation mechanisms of microbial consortia require a better understanding to optimise bioremediation conditions and predict/control the outcome of bioaugmentation. Studies of complex adaptive systems have previously revealed characteristic behaviours such as resilience and regime shifts. Hosoda et al. [56] proposed a population–reaction model based on a combination of microbial experimental ecosystems and a hierarchical dynamic model.

In our earlier study, it was hypothesised that eight bacterial cultures belonging to the *Pseudomonas* spp. and *Stenotrophomonas maltophilia* groups, originating from distinct soil samples of the same oil-contaminated site and representing a close phylogenetic relationship, could considerably increase their activity by serial batch cultivations (seven days each) with a stepwise increase (1%, 3%, 5% *w*/*w*) in diesel oil concentration [57]. The inoculum was prepared by mixing aliquots of the eight individual strains, which were pre-grown in a Bushnell-Haas (BH) broth supplemented with 0.5% molasses for 24 h and justified by optical density, thus achieving an equal initial cell concentration of each isolate at the beginning of the cultivation. The overall count of colony-forming units (CFUs) and enzyme activity in cultures was higher in the presence of 0.5% molasses as compared to that with 0.05% molasses, irrespective of the diesel oil concentration and the step of the serial batches. In the sets with 0.05% molasses, the highest cell biomass was detected after step 2, i.e., in the presence of 3% diesel oil. Visual changes in culture turbidity and colour indicated the formation of biosurfactants after just 48 h of incubation. The negative effect of the serial batches with increasing diesel oil concentrations from 1% to 5% was detected for fluorescein diacetate hydrolysis activity at 0.05% molasses, and the same was detected for dehydrogenase activity at both molasses concentrations tested. Conversely, urease activity was shown to increase gradually with increasing diesel oil concentrations in the broth amended with 0.5% molasses in three serial batches [57].

The adaptation of inoculum has recently been described by [58] for nitrifiers at enhanced concentrations of ammonium (1.6–150 mgN/L/d) and NaCl (2–30 g/L). Ammonium-oxidising bacteria prepared with 15 and 30 g/L salinity demonstrated a higher resistance to salinity changes than ammonium-oxidising archaea and comammox. The authors recommend separately preparing cultures for freshwater (low salinity) and brackish marine uses [58].

In another study, the application of aqueous aerated extracts from a biomixture acclimated with ibuprofen, diclofenac and triclosan was examined. The dissipation of 90% of the diclofenac and triclosan required 60 and 108 days less, respectively, than did the controls [42].

The amount of inoculum is another important factor which can influence biodegradation performance. According to the data reviewed by [59], the inocula concentrations can range from 10^7^–10^9^ CFU/mL for the enhanced bioremediation of diesel–biodiesel-polluted soils. Other studies have reported that an increased microbial activity did not always result in effective degradation [59].

## 5. Application of Immobilised Cells

Planktonic bacteria are sensitive to environmental variations, thereby limiting their application. Bioaugmentation in both drinking water treatment reservoirs and biological filters can be challenged by the washout/loss of bioaugmented bacteria. Numerous new immobilization methods have been developed to solve problems associated with the application of enzymes, such as directional immobilisation, co-immobilisation of multiple enzymes, and new immobilisation carriers [9]. Among promising carriers for biofilm development, biochar is an excellent carrier with potential for decontamination and bacterial adsorption via rich functional groups, a large specific surface area, high porosity, and excellent biocompatibility. Microbes can be immobilised on biochar via different fixation methods such as adsorption, entrapment, cross-linking, and covalent bonding or a combination of two methods [31,60]. Saeed et al. [45] recently reviewed advances in biochar and plant growth-promoting rhizobacteria engineering systems for hydrocarbon degradation and showed that the synergistic effect of biochar and PGPR depends on the concentration of biochar and PGPR used [45]. Recent studies which have applied immobilised cells for biodegradation processes are summarised in Table 2.

The use of quorum sensing (QS) in a biofilm reactor has recently gained significant research interest due to its vital role in biofilm formation, especially acyl-homoserine lactone (AHL) [61]. However, it is not economical or practical to regulate QS in a biofilm reactor through dosing pure AHL. Dosing AHL-producing bacteria might instead be more reasonable [61]. Therefore, in the context of QS, bioaugmentation approaches are also very important.

**Table 2:** Engineered materials for improvement in biofilm performance and in the shelf-life of desiccated microbial strains.

Carrier, Technique	Microorganism	Effect	Reference
Shell encapsulation	*Bradyrhizobium*	Protector: albumin-trehalose. Stabilized desiccated cells for 4 months at high relative humidity, increased the glass transition temperature	[62]
Bark biochar	Activated sludge	Vertical flow mesocosm-constructed wetland. Removal (>40%) of irbesartan and carbamazepine	[63]
Biochar	10% adapted thermophilic *Methanosarcina thermophila* on 2 g/L biochar	Anaerobic digestion. Methane yield increased up to 35%	[64]
Perlite	10^10^ CFU/g	Anaerobic digestion. Biodegradation of olive cake	[65]
Fe-modified zeolite grains	10^10^ cells/g of biocomposite (selected microbial consortium)	Soil microcosm. 80% herbicide MCPA (2-methyl-4-chlorophenoxyacetic acid)	[66]
High-density polyethylene (HDPE) carrier	*Sphingomonas rubra* BH3T as a quorum-sensing bacteria	Moving bed biofilm reactor (MBBR) at 5 °C. Higher chemical oxygen demand and NH4 +-N removal rate (93% and 75%). Co-culture with Nitrospira. The increased biofilm thickness (60.23%) during the whole operating time, accompanied by more potent adhesion force (61.59%), was related to increased polysaccharides and proteins in the biofilm	[61]
Gel-immobilization enhanced with biochar	Ammonia-tolerant methanogens	Continuous biogas reactor. Long-term ammonia resistance	[67]
Aerobic granules with microalgae	Aerobic granular sludge from aquaculture WW	Continuous flow granular reactors. Removed up to 77% and 80% of ammonium and nitrite	[68].
Algal organic matter (AOM) and humic substances (HS)	Injection of contaminant-degrading microorganisms	Decrease in removal of cyanotoxins like microcystin-LR (MC-LR). Decreases in MC-LR biodegradation rate of 1.6- and 3.4-fold in the presence of AOM and HS, respectively	[69]

## 6. Bioaugmentation Strategy

It is important to note that bioaugmentation schemes, which are described in different studies, can be reproducible but are sometimes not comparable because of differences in experimental setup, feedstock/soil/water composition, measurement units, the physiological state of the inoculum, etc. Nevertheless, rigorous analyses of various bioaugmentation experiments should be useful for choosing the most suitable treatment scheme.

The dosing ratio and dosing time play a vital role in the coordination and adaptive potential of the functional flora in the bioaugmentation system. The dosing strategies for the bioaugmentation of seafood processing WW in a sequencing batch bioreactor using artificially constructed mixed bacteria systems have been described by [2]. The reactor was operated with an 8 h cycle including 5 min of settling, 5 min of decanting (a volumetric exchange ratio of 50%), 5 min of filling, 105 min of anaerobic reaction, and 360 min of aerobic reaction. The use of a bacterial agent as a dosing compound in the batches (supplementing 2.5% on day 1 and day 10, respectively) dramatically increased the removal of NH4+-N and total nitrogen of seafood processing WW in winter from 66 89% and 52.77% to 79 0.02% and 69.97%, respectively [2].

The performance of anaerobic digestion also depends on the bioaugmentation dosage. Thus, the optimal dosage was determined to be 0.27 g VS_bioaugmentation seed_/g VS_chicken manure_, which could be adopted for rapid start-up or improving a continuous digester for treating chicken manure. Higher bioaugmentation doses (0.34 g VS_BS_/g VS_CM_) did not exhibit a significantly improved bioaugmentation efficiency [70].

The effects of single and routine bioaugmentation with *Methanosarcina thermophila* combined with the addition of biochar in the anaerobic digestion of food waste were compared by [71]. Specifically, 10% *v*/*v* of the microbes grown on biochar (1 g/L) were added during the setup of the reactors, which is in contrast to a routine bioaugmentation wherein the same amount of supplements were added over ten feeding cycles. The best routine reactor showed 37% more yield, while the best single reactor presented 32% [71].

The effect of bioaugmentation with exogenous activated sludge on the nitritation–anammox process in a sequencing batch reactor was studied by [44]. Two bioaugmentation strategies were tested: the exogenous sludge was added either immediately after the inoculation with the anammox activated sludge or when a stable mode of nitrogen removal was achieved. The authors reported a positive effect of bioaugmentation when carried out either at the launching of a bioreactor (a 15% increase in nitrogen RE) or after its long-term operation (a 21–35% increase in nitrogen RE); it had a short-term effect and should be used carefully [44].

Regarding the activation of the biodegradation process by nutrients/stimulants, [9] recently reviewed the emerging trends for the enhancement of co-metabolism for pollutant degradation efficiency [9]. In the case of Fenton oxidation and bioremediation of oil lubricant-contaminated soils, the addition of citrate or citric acid led to the further oxidation of the oil pollutant by forming chelation and preventing ferric oxide precipitate production. On the other hand, ammonium chloride and monosodium glutamate served as biostimulants that fostered the growth of indigenous petroleum or hydrocarbon degraders [72]. The addition of methylated β-cyclodextrin for bioavailability enhancement was previously proposed by [25] in a study of the degradation of chlorpyrifos from soil. The biostimulation effect of neat biodiesel was recently reviewed by [59]. It was also shown that rhamnolipids enhance pyrene bioaugmentation as a carbon source and as a biosurfactant, stimulating more active pyrene degraders and reconstructing microbial communities [73].

Biostimulation can result in an even higher RE than bioaugmentation. Thus, over a 90-day experiment (7.5 L bioreactors), the biodegradation of 35 mg/kg benzo(a)pyrene (BaP) and 28 mg/kg dichlorodiphenyltrichloroethane (DDT) was more efficient in the set employing biostimulation only, as compared to the one using bioaugmentation. However, bioaugmentation resulted in a toxicity drop of 90%, while this value was only 48% for the biostimulation set [74].

The addition of natural sorbents, e.g., minerals (zeolite, kaolinite, vermiculite, diatomite), organics (peat), carbonaceous (biochar) materials, and mixed sorbent (consisting of granular activated carbon and diatomite), to soils contaminated with crude oil resulted in a reduction in soil toxicity, decrease in soil hydrophobicity, optimization of soil pH and of the water–air regime, thus considerably stimulating the oil degradation [75].

Gibert et al. [76] studied the efficiency of nano zero-valent injection pulses for the removal of nitrate and pesticides (dieldrin and lindane) in continuous-flow packed columns promoted by the addition of acetate and/or an inoculum rich in denitrifiers. The combination of heterotrophic denitrifiers and abiotic chemical nitrate reduction promoted by the pulse injection of zerovalent iron nanoparticles (nZVI) resulted in up to 99% removal of NO_3_^-^. The removal of the target pesticides occurred not due to biodegradation, but via adsorption onto the soil or chemical degradation by nZVI [76].

**Figure 1:**
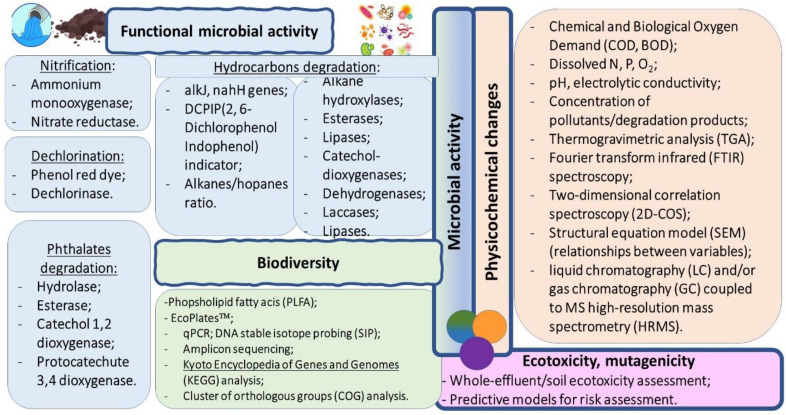
Biomonitoring parameters for assessing the performance of a bioremediation process [16,54,74,77,77,78].

The biomonitoring parameters used for assessing the performance of a bioremediation process are summarised in Figure 1.

## 7. Conclusions

In order to improve bioaugmentation performance, researchers mainly need to solve the problems of long-term residence of special functional bacteria and maintenance of multi-bacteria interactions [2]. If the total breakdown of the remaining contaminants is not achievable, immobilising and reducing the bioavailability of organic pollutants in soils is critical. Monitoring microbial activity on a regular basis by using methodologies for controlling problematic volatile organic compounds, ecotoxicity, pollutant leaching, etc., is necessary [79]. Multi-compound pollution, new findings in inoculum conservation for commercial uses, and the further development of process monitoring will be the main topics for further research in the field of bioaugmentation-assisted bioremediation.

## Figures and Tables

**Table 1:** Studies on biodegradation with the application of *Bacillus* spp., *Pseudomonas* spp., and *Rhodococcus* spp. as bioaugmentation agents.

Culture for Bioaugmentation		Type of Contaminant	Removal Efficiency (RE), Environment, Co-Cultures	Reference
Genus	Species/ Strain			
*Bacillus*	*B. megaterium* and *B. safensis*	Chlorpyrifos (CLP)	RE in solution: 99% of CLP in a solution with an initial concentration of 10 mg/L after 60 days. RE in soil: 61–65% after 100 days.	[25]
	*B. firmus*	Phenanthrene	RE: 1.73 mg/kg soil day for 56 days. Soil, anaerobic nitrate-reducing environment.	[26]
	*B. subtilis*, *B. licheniformis*, *B. megaterium*, *B. cereus*	Nitrogen in WW	RE: 92% of nitrate at the laboratory and 62% outdoors. A plug flow system; *Arthrobacter* sp., *Acinetobacter parafneus*, *Corynebacterium* sp., and *Streptomyces globisporus*	[27]
	*B. megaterium*	Nitrogen in WW	RE: 62.76% after 29 d. Secondary salinized soil, pot experiment. Improved NO_3_^-^ removal rate.	[28]
	*Bacillus* sp.	Decachlorobiphenyl	RE: up to 10.51% after 21 days. Initial conc. 200 mg/kg soil. *Staphylococcus* sp. and *Acinetobacter* sp. Consortia better than individual strains.	[29]
	*Bacillus* spp. and *B. aryabhattai*	Aquaculture WW	Algal–bacterial bioflocs and microbe–rice bran complexes, *Scenedesmus dimorphus* and *Chlorella* sp. 1:1	[30]
	*B. paramycoides*	Sulfamethoxazole (SMX) and Zn^2+^	Biochar-immobilized. After five rounds of reuse, RE: 43.24% for SMX and 50.34% for Zn^2+^	[31]
	*B. safensis*	Secondary composting	Indoleacetic acid (IAA)-production, assimilation of soluble salt, condensation and aromatization of humus, accumulation of dissolved organic nitrogen and carbon. *Corynebacterium stationis* subsp. safensis.	[32]
*Pseudomonas*	*Pseudomonas* sp.	Sulphide	RE: two-fold increase of elemental sulfur generation. Establishment of a stronger biofilm structure. Granular sludge bed reactor. *Arcobacter*, *Azoarcus*	[33]
	*P. mendocina, P. putida*	Nitrogen removal in petroleum WW	RE: 92.4% of NH4 +-N; 79.8% of total nitrogen. Heterotrophic nitrification, aerobic denitrification. *Brucella* sp., *Paracoccus* sp.	[34]
	*P. plecoglossicida*	Crude oil	76.7% ability to degrade crude oil in a liquid broth. 14 days.	[11]
	*P. stutzeri*	Glyphosate	RE: 53% and 79% for synthetic and real WW with 5 mg/L glyphosate. Continuous photobioreactors. Acclimation to glyphosate from 5 to 50 mg L-1. *Comamonas odontotermitis*; *Sinomonas atrocyanea*, *Chlorella protothecoides*.	[35]
	*Pseudomonas* sp.	Benzene, toluene, ethylbenzene, and p-xylene (BTEX)	RE: 20 mg/L in a mineral salt medium for 6 h. *Variovorax paradoxus*	[36]
	*P. songnenensis*	Oil-contaminated desert soil	RE: 73.6%, 69.3%, 50% and 50% in soils polluted with 1%, 10%, 20% and 30% oil, respectively, after 6 months. *Actinotalea ferrariae*, *Arthrobacter ginsengisoli*, *Dietzia cinnamea*, *Dietzia papillomatosis*	[37]
	*Pseudomonas* sp.	Fluoride	RE: 77.54%, 99.39%, and 67.25% for F^-^, NO_3_^-^, and Ca^2+^, respectively, for 8 h. Microbially induced calcium precipitation (MICP). Self-assembled fungus-flexible fibre composite microspheres. *Phoma* sp.	[38]
*Rhodococcus*	*R. ruber*	Chloroxylenol (PCMX)	RE: 97% during 22–30 cycles. 25 mg/L PCMX within 180 min in activated sludge system. Aerobic 100 mL batch, 4% inoculum (*v*/*v*). *R. ruber* became dominant within 30 cycles. Detoxify nitrification system.	[19]
	*R. ruber*	Quinoline and its toxic intermediate 2-hydroxyl quinoline (2-HQ)	RE: for quinoline, 100% for 1.5 h. For 2-HQ, max. appearance of 0.025 mM within 0.5 h, 100% degradation after 1 h. Aerobic 100 mL batch, 15% inoculum (*v*/*v*), initial concentration of quinoline of 0.25 mM. Accelerated nitrification and enriched *Nitrospira*.	[20]
	*R. erythropolis* (Genetically engineered expressing *Nirs* and *AMO* (rRho-NM).)	Nanofiltration concentrate of landfill leachate	RE: total organic carbon 63–89%; NH4–N 72–82%; total nitrogen 63–87%; chemical oxygen demand 81–95%, dependent on treatment mode. Integrated system of advanced oxidation processes cooperated with rRho-NM. Bioaugmentation to the aerobic fluidized reactor (2 L), inoculum 10^6^/mL.	[21]
	*R. pyridinivorans*	Di-(2-ethylhexyl)phthalate (DEHP)	RE: 89.94% of DEHP within 84 h. Initial DEHP conc. 5 mg/L DEHP in 10 mL municipal WW (batch). Aerobic denitrifying phosphate-accumulating bacterial strain RL-GZ01.	[39]
	*R. biphenylivorans*	Biphenyl and polychlorinated biphenyl (PCB) 31	RE: biphenyl 96% within 5 days, PCB31 92% for 3 days. Initial conc. biphenyl 500 mg/L, aerobic 5 mL batch cultivation, pH 7.0.	[40]
	*Rhodococcus* sp.	3-Methylindole (skatole)	RE: >99% for 24 h. Initial skatole conc. 60 mg/L. Degradation performance in consortium. Inoculum 2%.	[41]

## Data Availability

Not applicable.
